# Improvements in medical quality and patient safety through implementation of a case bundle management strategy in a large outpatient blood collection center

**DOI:** 10.1097/MD.0000000000010990

**Published:** 2018-06-01

**Authors:** Shuzhen Zhao, Lujia He, Chenchen Feng, Xiaoli He

**Affiliations:** West China Hospital of Sichuan University, Chengdu, Sichuan Province, China.

**Keywords:** case bundle, medical quality, patient safety

## Abstract

Laboratory errors in blood collection center (BCC) are most common in the preanalytical phase. It is, therefore, of vital importance for administrators to take measures to improve healthcare quality and patient safety.

In 2015, a case bundle management strategy was applied in a large outpatient BCC to improve its medical quality and patient safety.

Unqualified blood sampling, complications, patient waiting time, largest number of patients waiting during peak hours, patient complaints, and patient satisfaction were compared over the period from 2014 to 2016.

The strategy reduced unqualified blood sampling, complications, patient waiting time, largest number of patients waiting during peak hours, and patient complaints, while improving patient satisfaction.

This strategy was effective in improving BCC healthcare quality and patient safety.

## 1 Introduction

The primary site for patient care is the outpatient clinic.^[1]^ In 2011, Chinese hospitals were responsible for approximately 35% of all outpatient clinic visits. ^[2]^ It is not surprising, then, that outpatient clinics are crowded and staff have heavy workloads. This is especially true in large general hospitals. Laboratory testing is one of the most common procedures in outpatient care, and it was reported to affect 60% to 70% of the most important decisions on medication, admission, and discharge. ^[3]^ Testing errors cause unnecessary investigations or inappropriate treatment, as well as dissatisfaction with medical services. ^[4]^

Laboratory errors can be categorized as preanalytical, analytical, or postanalytical. ^[5]^ Most often, errors occur in the preanalytical phase. ^[6,7]^ In a teaching hospital in Tehran, Iran, Abdollahi et al^[8]^ reported an overall laboratory error rate of 6.3%, with 65.1% of those errors occurring in the preanalytical phase. Similarly, in a veterinary clinical laboratory, Hooijberg et al^[5]^ reported preanalytical errors ranging from 52% to 77%, analytical errors from 4% to 14%, and postanalytical errors from 9% to 21%. As the preanalytical process takes place mainly in blood collection center (BCC), sample quality is extremely important.

Additionally, patients’ feelings, experiences, and satisfaction regarding medical care are becoming increasingly important indicators of the quality of medical care.^[9]^ For those requiring outpatient phlebotomies, the long process of registration, waiting for a doctor, and payment can result in impatience and a lack of satisfaction. Patient satisfaction can be further reduced by poor blood collection environments and bad medical experiences, not to mention unqualified samples (e.g., those with hemolysis or blood clotting) or local hematomas that may cause suffering.

Measures such as regular monitoring, staff education, ^[10,11]^ and preanalytical workstations ^[12]^ have been implemented to improve sampling quality and reduce testing errors. However, the quality of care and patients’ satisfaction in BCC have not been paid enough attention. In 2001, the concept of “care bundles” was first proposed in a white paper by the Institute for Healthcare Improvement (IHI). The white paper recommended a set of evidence-based interventions for patients and provided a set of recommendations for improvements in the environment of the outpatient clinic. These interventions and recommendations separately improved patient outcomes, and when implemented together, resulted in markedly improved outcomes. ^[13,14]^ Case bundle strategies have been widely developed and applied in medical quality management. ^[15–17]^ Patient safety is improved and guaranteed by these strategies, which include leadership walkarounds, structured educational programs, team-based strategies, and multicomponent organizational interventions. ^[18,19]^ To our knowledge, case bundle strategies have not been applied in BCC. To improve medical quality and guarantee patient safety, we implemented a case bundle management strategy in West China Hospital (WCH)'s BCC in January 2015. Besides some commonly used strategies such as staff training, formulation of procedures, and regular monitoring, innovative strategies such as changing the attribution of nurses in the BCC, the introduction of part-time nurses, and arranging blood collection via WeChat were also applied. This bundle management strategy demonstrated significant improvements in medical quality and patient safety.

## 2 Methods

### 2.1 Setting

The study was conducted in a large outpatient BCC at WCH, Chengdu, China. In 2014, there were over 16,000 outpatients daily, with more than 3000 patients providing blood samples each day. The BCC obtained 1.6 million blood samples over the course of the year. Our hospital has a large number of outpatients, contributing heavy workloads to the BCC. The situation is similar in other BCCs in China.

### 2.2 Population and design

We implemented the case bundle management strategy in January 2015. In December, data on the quality of blood samples, incidence of blood collection complications, complaint rate, and patient satisfaction were recorded and sorted for 2014, 2015, and 2016. Average waiting time and the largest number of waiting patients during peak hours were collected for September 2014, 2015, and 2016. September was chosen because the largest number of patients were seen in that month.

### 2.3 Case bundle management strategy

#### 2.3.1 Clarification of nursing staff attribution

Currently, there is no consensus regarding staff work processes or attribution for BCCs in China or the rest of the world. Clear, evidence-based exploration of these issues is therefore essential. Before January 2015, blood sampling nurses at WCH worked within the Department of Experimental Medicine. The management model before 2015 focused on analytical and postanalytical processes, while the service standards, practice norms, standardized sample handover, and health education and training of nurses received less attention. This resulted in poor preanalytical care quality and low patient satisfaction. Since the implementation of the case bundle management strategy in 2015, the BCC has been housed within the Outpatient Department, and nurses in the BCC are now part of the Nursing Department.

#### 2.3.2 Personnel increase for the BCC

The Nursing Department allocated 10 additional nurses to the BCC, increasing the total number of nurses to 32. Simultaneously, full- and part-time work was combined, with flexible scheduling implemented by the recruitment of several registered nurses (RNs). After training, these newly recruited nurses worked as part-time blood sampling staff during peak hours (from 7:00 am to 11:00 am).

#### 2.3.3 Space management

In 2015, there were 4000 daily patients for blood collection. Before the application of case bundle management strategies, there were up to 74 patients waiting during peak hours, with the mean waiting time being almost 120 minutes. The original 18 service windows did not meet demand; therefore, an additional 9 windows were added. In addition, staff distributed leaflets to patients during peak hours, directing them to another BCC with fewer patients waiting.

#### 2.3.4 Arranging blood collection via WeChat

By prior reservation using the WeChat smartphone application, patients were provided with alternative times for blood collection.

#### 2.3.5 Improving nurse training, increasing service awareness, and implementing strict blood collection standards

Venous blood collection protocol was as follows: consult standards and check regulations; assess whether the patient meets the requirements for blood collection, (e.g., fasting); assess the patient's blood vessels; conduct the collection process in accordance with established operating procedures; offer health education after blood collection, including correct application of pressure, duration of pressure application, as well as the time and place for access to blood collection reports.

Code of conduct for blood sampling nurses, including what to say, how to act, and how to implement appropriate service standards. The following code of conduct for “what to say” was developed for phlebotomy nurses: ask patients to show blood collection certificate and barcode; confirm the patient's name, age, and last meal time if fasting was required; provide blood vessel education; inform the patient of the possibility of discomfort due to skin puncture; remind the patient not to touch disinfected skin, and instruct the patient to make a fist; tell the patient to maintain puncture site pressure for 5 minutes after blood collection without bending the arm; ask the patient to take proper custody of their certificate and inform them of the time and place for receipt of their blood collection report.

The following code of conduct for “how to act” was developed for blood sampling staff: check the identity of the patient and other pertinent information; expose the blood sampling site; assess blood vessels to determine an appropriate puncture site; recheck pertinent information; place a glove on each hand, tie the tourniquet, and conduct skin disinfection until the skin is dry; perform venipuncture; pull the needle; assist the patient in applying pressure to the puncture site; give the patient the certificate for the collection report; collect the used medical equipment, remove gloves, and disinfect hands.

The following service standards were developed: always present a kind, active, and enthusiastic attitude; be responsive to the patient; dress properly; follow standards of practice; and collect qualified samples.

#### 2.3.6 Complication reduction by enhancement of blood collection education

A health education video exhibiting blood collection notices was screened. Reminder cards providing guidance on diet before blood collection were posted in each sampling service window. Advice regarding constrictive clothing that could cause subcutaneous hematomas was provided. After blood collection, nurses guided patients on the proper application and duration of pressure at the venipuncture site, ensuring that the patient understood venipuncture.

#### 2.3.7 Blood sample “handover rules”

Before this case bundle management strategy was implemented, samples were directly collected from the blood collection window by personnel from the Experimental Medicine Department, where sample handover rules were inconsistent. If a problem arose, responsibility was unclear. To ensure clear responsibility and avoid sample loss, a third party was authorized to participate in sample transfer. The specific process was as follows. After blood sample collection, nurses quickly scanned barcodes, checked computer information, and sorted samples. The third party rescanned barcodes, and then input the encoded information into the computer. This was the first handover. Next, the third-party personnel transported the blood samples to the medical laboratory, where the handover was completed face to face.

### 2.4 Variables

#### 2.4.1 Sample quality

Unqualified samples included those that were hemolyzed, coagulated, or had barcode errors. The unqualified sample rate was defined as the sum of the unqualified samples/total number of samples × 100%. Six Sigma methodology was also used to measure sample quality. Values lower than 3σ indicated that the sample quality did not meet clinical requirements; those higher than 3σ but lower than 4σ indicated that sample quality should be enhanced; those higher than 4σ were defined as good quality; and those scoring 5σ were considered excellent quality.^[14–17]^

#### 2.4.2 Blood collection complications

These included subcutaneous congestion, hematoma, infection, fainting, or nerve damage. The mean complication rate was calculated as the number of patients who had complications/total number of blood collection patients × 100%.

#### 2.4.3 Mean waiting hours and largest number of waiting patients during peak hours

Peak blood collection hours were usually from 7 am to 11 am (primarily because of the need for fasting before blood collection). During workdays from September 2014 to 2016, a researcher instructed patients waiting between 7 am and 11 am to use their phones to keep track of their waiting time. Patients then showed the researcher the recorded time before undergoing blood collection. To record the largest number of waiting patients, a researcher would count the number of patients in line every 30 minutes from 7 am to 11 am.

#### 2.4.4 Complaint rate and overall satisfaction

Oral or formal complaints from patients or their family members were recorded. An Outpatient Nursing Satisfaction Questionnaire consisting of 12 items including the nurse's appearance, use of civilized language, and a rating of the outpatient clinic environment was developed. Satisfaction was rated on 5 levels: very satisfied, satisfied, general, not satisfied, very dissatisfied. The Weighted Satisfaction Number was defined as the sum of very satisfied × 1, satisfied × 0.9, general × 0.5, not satisfied × 0.2, very dissatisfied × 0. Overall satisfaction was calculated as: Weighted Satisfaction Number/Total items × 100%. The questionnaire's validity was 0.85. The test–retest reliability was 0.93, determined during a 2-week interval for 20 patients. A total of 353 questionnaires were distributed in 2014, 352 in 2015, and 358 in 2016.

### 2.5 Ethical considerations

The study was approved by the Ethics Committees of West China Hospital of Sichuan University (number 139). Patients were informed about the aims and procedures of the study and gave oral consent when they filled the questionnaire. As the investigation of patient satisfaction is a monthly BCC task, the Ethics Committees of West China Hospital of Sichuan University granted exemption from informed consent.

### 2.6 Statistical methods

SPSS software (version 17.0; SPSS Inc, Chicago, IL) was used for analysis. A *χ*^2^ test was used to compare rates, and the Student *t* test and rank-sum test were used for 2 sample comparisons. *P* values < .05 were considered statistically significant.

## 3 Results

### 3.1 Disqualified samples in the preanalytical phase

The total number of samples showed an increase from 2014 to 2016. Hemolytic sample σ values increased from 4.25 to 4.69, clotting sample σ values increased from 4.89 to 5.2, and incorrect barcode σ values increased from 4.85 to 5.14. The unqualified sampling rate decreased from 0.366% in 2014 to 0.095% in 2015, and 0.102% in 2016 (Table 1).

**Table 1:**
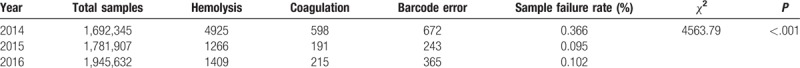
Details of unqualified samples in 2014, 2015, and 2016.

### 3.2 Complication rate, patients’ medical experiences, and overall satisfaction

The total number of patients undergoing blood collection increased from 872,460 in 2014 to 884,844 in 2015 and 1,019,257 in 2016. However, the mean complication rate decreased from 0.017% in 2014 to 0.003% in both 2015 and 2016 (Table 2). Patients’ medical experiences showed significant improvement. The mean waiting time during peak hours decreased from 120 to 20 minutes in 2015, and to 18 minutes in 2016 (Table 3). The largest number of waiting patients during peak hours reduced from 74 in 2014 to 15 in 2015 and 17 in 2016 (Table 3). Patients’ complaint rate decreased from 0.008% in 2014 to 0.002% in 2015, and 0.003% in 2016 (Table 4). As for satisfaction, the number of valid Outpatient Nursing Satisfaction Questionnaires was 344, 350, and 350 in 2014, 2015, and 2016, respectively. Patients’ overall satisfaction levels increased from 75.91% in 2014 to 97.35% in 2015 and 97.45% in 2016 (Table 5).

**Table 2:**
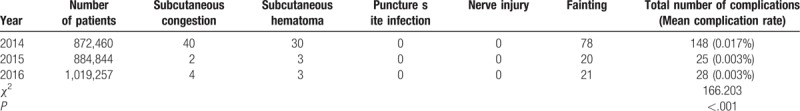
Details of blood collection complications in 2014, 2015, and 2016.

**Table 3:**
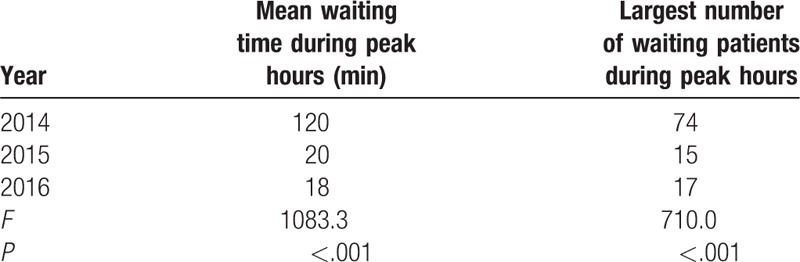
Mean waiting time and largest number of waiting patients during peak hours in 2014, 2015, and 2016.

**Table 4:**
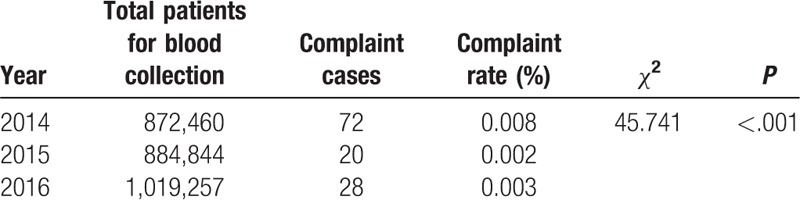
Complaint rates in 2014, 2015, and 2016.

**Table 5:**
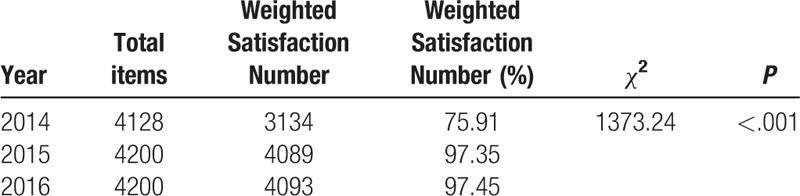
Patient overall satisfaction levels in 2014, 2015, and 2016.

## 4 Discussion

Laboratory testing, also called the total testing process (TTP), is a complex process. ^[12]^ It is usually divided into preanalytical, intra-analytical, and postanalytical phases. ^[5]^ Errors occurring in the preanalytical phase may account for up to 75% of total laboratory errors.^[4]^ The preanalytical phase involves physicians, patients, nurses or phlebotomists, laboratorians, and many procedures such as data entry, patients’ preparation, specimen collection, and sample delivery. ^[20]^

The number of patients undergoing blood collection in our BCC increased from 2014 to 2016. In 2016, the total number of blood collection patients reached 1,019,257. Before the case bundling management strategy was implemented, the unqualified sample rate in our BCC was 0.366% in the preanalytical phase and the waiting environment was poor. Hallways were seriously congested and there were safety risks. Patients, and even nurses, often fainted, and patients were dissatisfied. Hence, there was an urgent need for improvement in the quality of medical care, as well as patient safety.

Although many strategies have been reported to improve laboratory results, ^[11,21,22]^ they focus mainly on sample quality, placing less importance on service standards and patients’ feelings and experiences. Therefore, case bundle management with an integrated strategy to improve sample quality, service quality, and patients’ experiences was implemented in WCH's BCC in January 2015.

Human error in medicine can from 3 ways: the person, the legal, and the system model. ^[23]^ We streamlined and systematized management procedures by considering the characteristics of the hospital and pinpointing specific issues, then summarized the problems that needed to be addressed: the past attribution of BCC ignored nurses’ care quality during blood collection, limiting the personnel input for BCC, laying a heavy workload on nurses and leading to poor medical experiences for patients; poor BCC environment needed improvement; and staff training, as well as strict sample handover rules, were required to enhance sample quality. In addition, various forms of patient education were required.

A sufficient number of RNs is critical for hospitals to provide high-quality medical care. ^[24]^ Hence, we increased personnel as the first step in addressing existing problems. Nurses in the BCC being part of the Nursing Department lays the foundation for the personnel input and hence, 10 additional full-time nurses were allocated to the BCC. The use of part-time nurses offers flexibility in filling RN shortage, ^[9,24,25]^ but little is known about their use in BCCs. In our study, part-time nurses from the inpatient ward were also chosen and trained. They worked during peak hours to reduce the workload of full-time nurses. Results indicated that this not only shortened patient waiting times but also improved the BCC environment. At the same time, human resources were fully utilized.

Service awareness was demonstrated to have a direct positive impact on patients’ first impression of the hospital.^[19]^ Improvements in the service attitude of nurses can reduce the incidence of nurse–patient disputes and increase patient satisfaction. ^[22]^ Although there was no consensus, our study did indicate that if the BCC nurses are part of the Nursing Department, their training and service awareness can be improved, as the Department of Experimental Medicine can pay more attention to sample analysis. What is more, the preanalytical process, which mainly includes data entry, patient preparation, specimen collection, and sample delivery, ^[20]^ all involved nurses. So, if the nurses in the BCC are part of the Nursing Department, this model could improve quality in that phase, but we still need to factor in the situation at every different facility.

To reduce waiting time and the number of patients during peak hours, we added several service windows and provided the option of scheduling blood collection via WeChat. Also, the staff distributed leaflets to guide patients to another BCC with fewer patients. As a result, the mean waiting time and the number of patients during peak hours were significantly reduced. In addition, the number of complaints decreased and overall patient satisfaction increased.

Developing clear written procedures and enhancing professional healthcare training were effective in preventing preanalytical errors. ^[26–28]^ To improve sample quality and reduce the complication rate of blood collection, we strengthened the training of nurses, implemented standard procedures, and enhanced patient education in various forms in the BCC. Strict sample handover rules were formulated and followed. In this way, the unqualified blood sampling and blood complication rates dropped, and communication between nurses and patients improved, leaving patients more satisfied with the BCC's service.

The current medical environment in China requires increasingly high standards of work and service in both medicine and nursing. Hospital administrators need to fully understand the characteristics of the hospital so that multifaceted management methods can be implemented. In this manner, the patient experience is optimized and the staff working environment improved by resource integration.

Limitations to this study exist. First, unqualified samples only included those that were hemolyzed or coagulated or had barcode errors. Other types of unqualified samples, such as those with insufficient volume or inappropriate containers, were not observed. However, these 3 constituted more than 95% of the unqualified samples in our BCC, so they were chosen as the indicators of sample quality. Second, results from a single hospital in China may not be representative of all other hospitals, so our study is more applicable to developing countries with huge populations and unbalanced medical resources.

## 5 Conclusions

The case bundle management strategy can be applied to improve healthcare quality and patient safety in a BCC. Nurses in BCCs being part of the Nursing Department have its advantage in managing service quality in the preanalytical phase. Part-time nurses can be hired to address the shortage of personnel in BCCs. New media like WeChat can be used to schedule blood collection and decongest the BCC.

## Acknowledgments

The authors thank Binwu Ying for his critical review of the manuscript with his permission to be named. The authors also thank the China Medical Board (0082827601130) and the Science and Technology Department of Sichuan Province (2013FZ0088) for funding this research.

## Author contributions

**Data curation:** Lujia He, Chenchen Feng.

**Funding acquisition:** Xiaoli He.

**Investigation:** Lujia He.

**Methodology:** Shuzhen Zhao, Xiaoli He.

**Project administration:** Shuzhen Zhao.

**Resources:** Shuzhen Zhao.

**Software:** Lujia He.

**Supervision:** Xiaoli He.

**Writing – original draft:** Shuzhen Zhao, Chenchen Feng.

**Writing – review & editing:** Shuzhen Zhao, Xiaoli He.
